# Atypical Presentation of Right Ventricular Cardiac Hamartoma in a Young Man

**DOI:** 10.14797/mdcvj.1158

**Published:** 2022-10-04

**Authors:** Ramez Barsoom, Lamees I. El Nihum, Qasim Al Abri, Areeba Ali, Susan L. Haley, Mohammed A. Chamsi-Pasha, Moritz C. Wyler von Ballmoos, Thomas E. MacGillivray, Michael J. Reardon

**Affiliations:** 1 Houston Methodist DeBakey Heart & Vascular Center, Houston Methodist Hospital, Houston, Texas, USA; 2 Texas A&M University School of Medicine, Bryan, Texas, USA; 3 Texas A&M College of Medicine, Bryan, Texas, USA; 4 Department of Pathology & Genomic Medicine, Houston Methodist Hospital, Houston, Texas, USA

**Keywords:** cardiac hamartoma, ventriculotomy, cardiac tumor, echocardiography, mass, right ventricle

## Abstract

Cardiac tumors in adults are exceedingly rare and usually benign. We describe a 29-year-old man with a previous diagnosis of interventricular septal hypertrophy who presented with increasing severity of dyspnea and fatigue. Work-up revealed a 4.9 × 3.7 cm mass at the base of the interventricular septum. Biopsy revealed a benign cardiac hamartoma atypically located in the right ventricle, and the mass was resected via right ventriculotomy.

## Introduction

Hamartoma of mature cardiac myocyte is a relatively recent term classified under benign tumor and tumor-like lesions of the heart. They often present a diagnostic challenge due to their nonspecific symptoms, which can include chest pain, fatigue, dyspnea, or palpitations. The present case has been highlighted due to its rarity, unique infiltrative radiological appearance, and poorly defined clinicopathologic spectrum of features.

## Case Report

A 29-year-old male with a previous diagnosis of hypertrophic cardiomyopathy presented to his cardiologist for episodes of fatigue, chest pain, and progressive dyspnea on physical exertion for the past 3 years, increasing in frequency and severity over the last 6 months. During this time, he experienced several episodes of ventricular tachycardia and one episode of near syncope. He was diagnosed with hypertrophic cardiomyopathy 4 years prior to presentation after a murmur was heard on routine exam, and it had been medically managed with a beta blocker.

**Figure 1:**
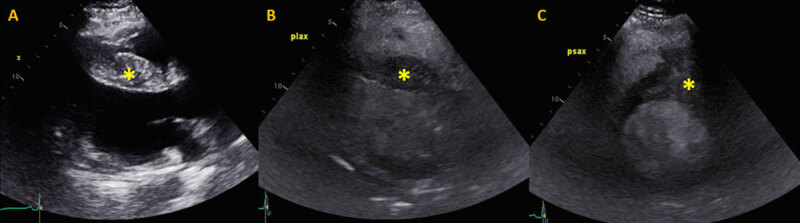
Preoperative transthoracic echocardiogram. **(A)** Transthoracic echocardiogram without contrast and **(B, C)** with intravenous contrast-enhancing agent. **(B)** Parasternal long axis view demonstrating asymmetric septal thickening/mass with contrast enhancement and **(C)** protrusion of the mass into the right ventricular outflow tract on parasternal short axis.

**Figure 2:**
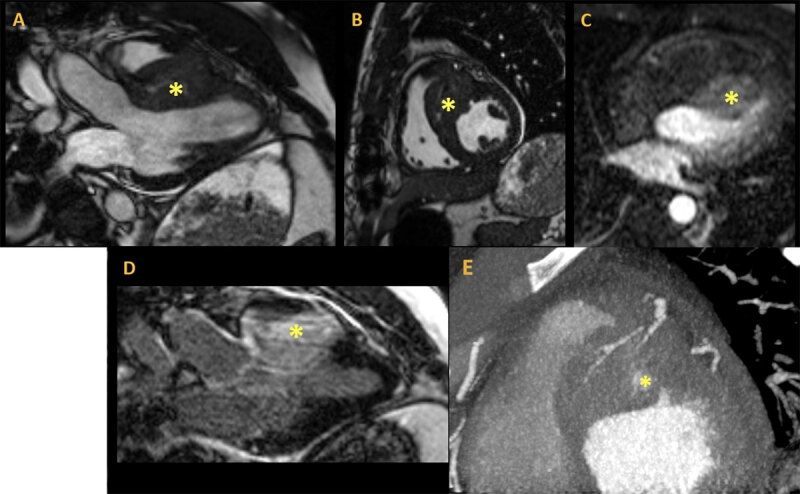
Preoperative cardiac imaging. Magnetic resonance imaging using steady state free precession cine sequence: **(A)** 3-chamber view, **(B)** short axis at the mid-level, **(C)**, first pass perfusion, and **(D)** delayed enhancement after gadolinium showing a large right ventricular (RV) mass measuring 4.9 × 3.7 cm invading the RV free wall, with **(E)** intense first pass perfusion and significant late gadolinium enhancement cardiac computed tomography demonstrating short axis basal anteroseptal mass protruding into the RV outflow tract with central contrast enhancement and multiple septal perforator feeding vessels.

Transthoracic echocardiogram revealed a prominent thickening at the base of the interventricular septum that extended into the right ventricular outflow tract (RVOT) (Figure 1 A-C). It was noncontractile and heterogeneous in appearance with a vascular component, suggestive of a tumor. The mass was confirmed on subsequent cardiac magnetic resonance imaging, which demonstrated a 4.9 × 3.7 cm mass extending from the anterior left ventricular wall to the inferoseptum and from the base of the heart through the mid-segment as well as into the RVOT (Figure 2 A-D). Cardiac computed tomography showed a basal anteroseptal mass protruding into the RVOT with central contrast enhancement and multiple septal perforator feeding vessels (Figure 2 E). At this time, the differential diagnosis included both benign and malignant etiologies.

A fluorodeoxyglucose positron emission tomography scan was then performed and demonstrated an area of hypermetabolism in the anterior aspect of the right ventricle medially. The maximum standardized uptake value (SUVmax) of this abnormality was difficult to assess due to the proximity of the left myocardium, which is normally and physiologically hypermetabolic. SUVmax of the lesion was approximated to be 7.0, concerning for the presence of malignant tissue. Given the vascular nature of the mass, a coronary angiogram and biopsy were performed in anticipation of surgical planning. Cardiac catheterization revealed normal coronary arteries and disorganized vascularization towards the septum. Right ventricular mass biopsy showed no neoplasia; however, there was some innocuous subendocardial fibrous tissue. Pathology report showed hypertrophic cardiomyocytes consistent with a cardiac hamartoma.

**Figure 3:**
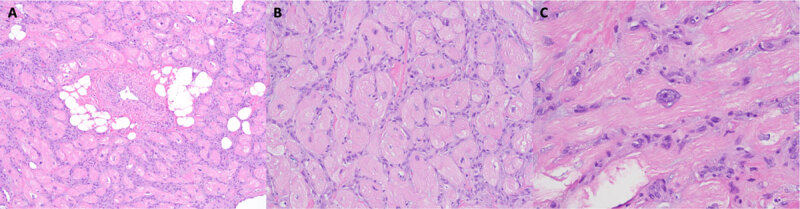
H&E stain at increasing magnification 100×. **(A)** The resected lesion was composed of disorganized, hypertrophic cardiac myocytes with interstitial fibrosis. **(B)** There were also scattered thickened intramural arteries, dilated venules, and small collections of adipocytes 200×. **(C)** At intermediate magnification, the myocytes were haphazardly arranged and enlarged with sarcoplasmic vacuolation and nuclear enlargement 400×. There were scattered enlarged nuclei, some with mild hyperchromasia, irregular nuclear contours, and inconspicuous nucleoli.

Due to significant symptoms with minimal exertion secondary to the mass causing RVOT obstruction, a palliative resection was performed via a right ventriculotomy. Postoperative pathology of the specimen showed a soft, tan, flesh-colored mass measuring 3 × 1.6 × 1.4 cm. Sections showed discrete lesions of marked myocyte hypertrophy with disorganization, focal scarring, and thickened intramural arteries (Figure 3 A-C).

## Discussion

Hamartomas consisting of mature cardiac myocytes are rare; as such, their clinicopathologic spectrum is not well defined in the literature. Based on a literature search, only 30 patients with a diagnosis of hamartoma of mature cardiac myocytes have been identified from 1998 to 2020 (Table 1).^1,2,3,4,5,6,7,8,9,10,11,12,13,14,15,16,17,18,19,20,21^ Of these 30, 19 were males (63%) and 11 were females (37%). The mean age was 32 ± 21 years, ranging from 6 months to 76 years. Eight of the 30 patients (27%) were identified in the pediatric population. The hamartoma was localized to the left ventricle in 13 patients, the right atrium in 7 patients, and the right ventricle in 3 patients; 2 patients showcased multiple locations. Ten of these patients (33%) were asymptomatic at time of presentation. The present case was an atypical presentation of hamartoma due to its location in the right ventricle rather than in the free wall of the left ventricle, which is the most commonly reported.

**Table 1:** Cardiac hamartoma literature search. A literature search revealed 30 patients with a diagnosis of hamartoma of mature cardiac myocytes from 1998 to 2020. The hamartoma was localized to the left ventricle in 13 patients, the right atrium in 7 patients, and the right ventricle in 3 patients; 2 patients showcased multiple locations. Ten of these patients (33%) were asymptomatic at time of presentation.^1-21^ CT: computed tomography; RVR: rapid ventricular response; WPW: Wolff-Parkinson-White.


YEAR PUBLISHED	AUTHOR	AGE	GENDER	CLINICAL SYMPTOMS	DIAGNOSTIC EVALUATION	TUMOR LOCATION	TUMOR SIZE

1998	Sturtz et al.	24	Male	Hypertension, palpitations, and premature contractions	—	Left ventricle	—

1998	Burke et al.	22	Male	Asymptomatic	Echocardiogram	Right ventricle	5 cm

1998	Burke et al.	28	Male	WPW syndrome and an episode of syncope	Echocardiogram	Right atrium	—

1998	Burke et al.	9	Male	Sudden death	Autopsy	Right atrium	1 × 2 cm

2001	Dinh et al.	33	Male	Generalized tachycardia	Echocardiogram	Left ventricle	4.5 × 3.1 × 4.4 cm

2004	Chu et al.	76	Male	History of hypertension	In surgery	Crista terminalis	0.5 × 1 × 0.5 cm

2005	Martínez QM et al.	33	Male	Palpitations and dyspnea	Echocardiogram	Left ventricle	4.5 × 5.5 cm

2008	Movahedi et al.	58	Male	Progressive dyspnea	In surgery	Right atrium	1.5 × 1 × 0.5 cm

2008	Fealey et al.	0.5	Male	Asymptomatic	—	—	—

2008	Fealey et al.	0.5	Female	Asymptomatic	—	—	—

2008	Fealey et al.	1.2	Male	Asymptomatic	—	—	—

2008	Fealey et al.	10	Male	Asymptomatic	Echocardiogram	Left ventricle	5 × 3 cm

2008	Fealey et al.	16	Female	Asymptomatic	Echocardiogram	Right ventricle	8 × 9 cm

2008	Fealey et al.	57	Male	Sudden death	—	—	—

2008	Fealey et al.	74	Male	Exertional dyspnea	—	—	—

2008	Menon et al.	16	Female	Weight loss	Echocardiogram	Right ventricle	8 × 7 × 3 cm

2008	Menon et al.	10	Male	Asymptomatic	Echocardiogram	Left ventricle	0.3 × 0.2 cm

2009	Hsu et al.	19	Female	Intermittent palpitations and dizziness	Echocardiogram	Left ventricle	4 × 7 cm

2009	Galeone et al.	56	Female	Asymptomatic	Chest CT	Pulmonary infundibulum	9 × 9 × 4 cm

2011	Dell’Amore et al.	35	Female	Palpitations and dyspnea	Echocardiogram	Left ventricle	4.2 × 3.3 × 2.7 cm

2013	Raffa et al.	41	Female	Chest pain	Echocardiogram	Right atrium	2.5 × 1.3 cm

2017	Ayoub et al.	14	Male	Asymptomatic	Echocardiogram	Left ventricle	9 × 5 × 6 cm

2017	Hadravská et al.	39	Female	Ruptured aneurysm and severe pneumonia	Autopsy	Left ventricle	4.5 × 3 × 3 cm

2017	Abuzaid et al.	21	Female	Chest pain and dyspnea	Echocardiogram	Left ventricle	1.6 × 1.3 × 1.9 cm

2017	Liu et al.	64	Male	Dyspnea	Echocardiogram	Left ventricle	2 × 3 cm

2018	Mantilla-Hernandez et al.	23	Female	Paroxysmal nocturnal dyspnea, RVR and edema	Autopsy	Right atrium	—

2018	Negri et al.	44	Female	Asymptomatic	Echocardiogram	Left ventricle	4 × 3 cm

2018	Kumari et al.	43	Male	Chest pain, orthopnea, and palpitations	Echocardiogram	Left ventricle	8 × 6 cm

2019	Fu et al.	41	Male	Cough, edema, dyspnea on exertion	Echocardiogram	Right atrium	2 × 1 × 0.5 cm

2019	Zhou et al.	41	Male	Dyspnea and edema	Echocardiogram	Right atrium	1.8 × 1.4 cm


## Conclusion

Hamartomas prove to be a diagnostic challenge due to nonspecific and often nonexistent symptoms. Given this as well as their slow-growing nature, surgical resection followed by postoperative microscopic pathological examination is the only reliable method of definitive diagnosis. However, microscopic features including myocyte hypertrophy and interstitial fibrosis in a whorled pattern are also nonspecific and characteristic of hypertrophic cardiomyopathy as well, which was the initial diagnosis of our patient. Distinction between hamartomas and hypertrophic cardiomyopathy on a small surgical or biopsy specimen has been proven difficult and inconclusive at times. Additionally, imaging studies alone are not pathognomonic for hamartoma. Thus, establishing a diagnosis requires a combination of clinical findings, imaging studies, and microscopic examinations.
